# Supplementary material for: Analysis of PPARγ Signaling Activity in Psoriasis
Source: Int J Mol Sci. 2021 Aug 10;22(16):8603. doi: 10.3390/ijms22168603 (PMC8395241; doi:10.3390/ijms22168603)
Supplement: Supplementary file 1 [file ijms-22-08603-s001.zip › Supplemental materials_Analysis of PPARg signaling activity in psoriasis/Pathway models/Models images and html files/PPARG inhibits phenotypes assotiated with psoriasis/142105.html]

cell adhesion


# Cell Process cell adhesion

|  |  |
| --- | --- |
| URN | urn:agi-gocellproc:0007155 |
| Total Entities | 139 |
| Connectivity | 8090 |
| Name | cell adhesion |
| ObjectType | Cell Process |

---

|  |  |
| --- | --- |
| ChildConcepts | trophoblast adhesion |
|  | astrocyte adhesion |
|  | basophil adhesion |
|  | fibroblast adhesion |
|  | osteoclast adhesion |
|  | osteocyte adhesion |
|  | pericyte adhesion |
|  | keratinocyte adhesion |
|  | mesangial cell adhesion |
|  | leiomyocyte adhesion |
|  | mast cell adhesion |
|  | myelocyte adhesion |
|  | myocyte adhesion |
|  | T-cell adhesion |
|  | sperm cell adhesion |
|  | chondrocyte adhesion |
|  | granulocyte adhesion |
|  | macrophage adhesion |
|  | monocyte adhesion |
|  | neutrophil adhesion |
|  | lymphocyte adhesion |
|  | thymocyte adhesion |
|  | platelet adhesion |
|  | blood cell adhesion |
|  | erythrocyte adhesion |
|  | neural stem cell adhesion |
|  | eosinophil adhesion |
|  | cell-matrix adhesion |
|  | glial cell adhesion |
|  | cell-cell adhesion |
|  | endothelial cell adhesion |
|  | epithelial cell adhesion |
|  | homophilic cell adhesion |
|  | heterophilic cell adhesion |
|  | neuron adhesion |
|  | leukocyte cell adhesion |
|  | cell anchorage |
|  | hemadsorption |
|  | bone marrow macrophage adhesion |
|  | regulatory T-cell adhesion |
|  | stem cell adhesion |
|  | Th17 cell adhesion |
|  | B-lymphoid precursor cell adhesion |
|  | marginal zone B-cell adhesion |
|  | haematopoietic stem cell adhesion |
|  | B-cell adhesion |
|  | dendritic cell adhesion |
|  | intermediate monocyte adhesion |
|  | myeloid derived suppressor cell adhesion |
|  | hematopoietic cell adhesion |
|  | activated CD4+ T-cell adhesion |
|  | erythroblast adhesion |
|  | classical monocyte adhesion |
|  | plasmacytoid dendritic cell adhesion |
|  | bone marrow stromal cell adhesion |
|  | CD4+ T-cell adhesion |
|  | umbilical vein endothelial cell adhesion |
|  | phagocyte adhesion |
|  | natural killer cell adhesion |
|  | antigen-presenting cell adhesion |
|  | peripheral lymphocyte adhesion |
|  | colony forming cell adhesion |
|  | immunocompetent cell adhesion |
|  | bone marrow derived mast cell adhesion |
|  | mononuclear leukocyte adhesion |
|  | granulocyte/macrophage progenitor adhesion |
|  | PBMC adhesion |
|  | epithelioid cell adhesion |
|  | immune system cell adhesion |
|  | inflammatory monocyte adhesion |
|  | T-lymphoid precursor cell adhesion |
|  | plasma cell adhesion |
|  | CD8+ T-cell adhesion |
|  | peripheral blood stem cell adhesion |
|  | peritoneal macrophage adhesion |
|  | microglia adhesion |
|  | Th1 cell adhesion |
|  | activated T-cell adhesion |
|  | immature B-cell adhesion |
|  | cytotoxic T-cell adhesion |
|  | bone marrow stem cell adhesion |
|  | capillary endothelial cell adhesion |
|  | megakaryocyte progenitor cell adhesion |
|  | erythroid cell adhesion |
|  | megakaryocyte adhesion |
|  | Langerhans cell adhesion |
|  | effector T-cell adhesion |
|  | burst forming unit E adhesion |
|  | Th2 cell adhesion |
|  | activated natural killer cell adhesion |
|  | double negative thymocyte adhesion |
|  | effector memory T-cell adhesion |
|  | germinal center B-cell adhesion |
|  | cancer stem cell adhesion |
|  | memory T-cell adhesion |
|  | helper T-cell adhesion |
|  | lymphoblast adhesion |
|  | activated B-cell adhesion |
|  | blast cell adhesion |
|  | activated monocyte adhesion |
|  | CD4+ memory T-cell adhesion |
|  | killer cell adhesion |
|  | bone marrow derived mesenchymal stem cell adhesion |
|  | foam cell adhesion |
|  | spleen lymphocyte adhesion |
|  | activated CD8+ T-cell adhesion |
|  | mononuclear phagocyte adhesion |
|  | cytotoxic lymphocyte adhesion |
|  | reticulocyte adhesion |
|  | neutrophil precursor adhesion |
|  | dermal microvascular endothelial cell adhesion |
|  | gammadelta T-cell adhesion |
|  | brain capillary endothelial cell adhesion |
|  | B-1 B-cell adhesion |
|  | prolymphocyte adhesion |
|  | bone marrow derived mononuclear cell adhesion |
|  | tumor-infiltrating lymphocyte adhesion |
|  | plasmablast adhesion |
|  | lymphokine-activated killer cell adhesion |
|  | lymphoid progenitor cell adhesion |
|  | myeloid progenitor cell adhesion |
|  | alveolar macrophage adhesion |
|  | naive T-cell adhesion |
|  | B-lymphoblast adhesion |
|  | conventional dendritic cell adhesion |
|  | follicular dendritic cell adhesion |
|  | mast cell precursor adhesion |
|  | central memory T-cell adhesion |
|  | lung fibroblast adhesion |
|  | CD8+ memory T-cell adhesion |
|  | sinusoidal cell adhesion |
|  | megakaryoblast adhesion |
|  | peritoneum mast cell adhesion |
|  | CD4+ naive T-cell adhesion |
|  | M1 macrophage adhesion |
|  | bone marrow derived dendritic cell adhesion |
|  | double positive thymocyte adhesion |
|  | cancer-associated fibroblast adhesion |
|  | CD8+ thymocyte adhesion |

---

|  |  |
| --- | --- |
| Pathway | Actomyosin-Based Movement |
|  | Cardiomyocyte Dysfunction |
|  | TRPM4/6/7/8 Signaling in Arterial Hypertension (Hypothesis) |
|  | Podocyte Dysfunction in Diabetic Nephropathy |
|  | Graves Ophthalmopathy |
|  | GNRH Neurons Development and Migration Impairment in Hypogonadotropic Hypogonadism |
|  | Genes with Susceptibility for Crohn's Disease and Ulcerative Colitis |
|  | Stomach Mucosal Layer Disruption |
|  | Macrophage Survival through CCR5 and CXCR4 Mediated Signaling |
|  | Osteoclasts Function in Osteopetrosis |
|  | Proteins Involved in Adhesion, Migration and Invasion of Endometriotic Cell |
|  | EPHA2 Mutations in Cataract |
|  | MYOC in Cell Adhesion in Glaucoma |
|  | Podocyte Dysfunction in Glomerulonephritis |
|  | FBLN5 Potentiates Migration and Proliferation of Endothelial Cells in Cancer |
|  | PPARG inhibits phenotypes assotiated with psoriasis |
|  | efects selected |
|  | Neighbors of cell adhesion |
|  | H2A-K119 and H3-K27 |
|  | Possible Interplay between SOX11, CCND1 and EZH2 in Mantle Cell Lymphoma |
|  | 1\_Osteoclast functional impairment in osteopetrosis |
|  | 1\_Formation of cysts in polycystic kidney disease |
|  | 1\_Dysfunction of cochlear hair cell stereocilia proteins in hearing loss |
|  | 3\_Cardiomyocyte dysfunction unrelated to cell junctions in ARVD |

---

|  |  |
| --- | --- |
| MedScan ID | 4107155 |

---

|  |  |
| --- | --- |
| Alias | cell adhesions |
|  | intercellular adhesions |
|  | cell adhesion |
|  | cell attachments |
|  | cell adherences |
|  | inter-cellular adhesion |
|  | cell cohesions |
|  | inter-cellular adhesions |
|  | cell surface} adhesion |
|  | adhesions of cell |
|  | adhesion of cell |
|  | cell contact |

---

|  |  |
| --- | --- |
| GO ID | 0007155 |

---
